# Evidence for Altered Basal Ganglia-Brainstem Connections in Cervical Dystonia

**DOI:** 10.1371/journal.pone.0031654

**Published:** 2012-02-22

**Authors:** Anne J. Blood, John K. Kuster, Sandra C. Woodman, Namik Kirlic, Miriam L. Makhlouf, Trisha J. Multhaupt-Buell, Nikos Makris, Martin Parent, Lewis R. Sudarsky, Greta Sjalander, Henry Breiter, Hans C. Breiter, Nutan Sharma

**Affiliations:** 1 Mood and Motor Control Laboratory, Massachusetts General Hospital, Charlestown, Massachusetts, United States of America; 2 Laboratory of Neuroimaging and Genetics, Massachusetts General Hospital, Charlestown, Massachusetts, United States of America; 3 Center for Morphometric Analysis, Massachusetts General Hospital, Charlestown, Massachusetts, United States of America; 4 Athinoula A. Martinos Center for Biomedical Imaging, Massachusetts General Hospital, Charlestown, Massachusetts, United States of America; 5 Department of Psychiatry, Massachusetts General Hospital, Charlestown, Massachusetts, United States of America; 6 Department of Neurology, Massachusetts General Hospital, Charlestown, Massachusetts, United States of America; 7 Department of Radiology, Massachusetts General Hospital, Charlestown, Massachusetts, United States of America; 8 Department of Neurology, Brigham and Women's Hospital, Boston, Massachusetts, United States of America; 9 Harvard Medical School, Boston, Massachusetts, United States of America; 10 Division of Graduate Medical Sciences, Boston University Medical School, Boston, Massachusetts, United States of America; 11 Division of Health Sciences and Technology, Massachusetts Institute of Technology, Cambridge, Massachusetts, United States of America; 12 Department of Psychiatry and Neuroscience, Faculty of medicine, Centre de recherche Université Laval Robert-Giffard, Université Laval, Quebec City, Quebec, Canada; 13 Department of Psychiatry and Behavioral Sciences, School of Medicine, Warren Wright Adolescent Center and Center for Self-Regulation, Northwestern University Feinberg, Chicago, Illinois, United States of America; The University of Western Ontario, Canada

## Abstract

**Background:**

There has been increasing interest in the interaction of the basal ganglia with the cerebellum and the brainstem in motor control and movement disorders. In addition, it has been suggested that these subcortical connections with the basal ganglia may help to coordinate a network of regions involved in mediating posture and stabilization. While studies in animal models support a role for this circuitry in the pathophysiology of the movement disorder dystonia, thus far, there is only indirect evidence for this in humans with dystonia.

**Methodology/Principal Findings:**

In the current study we investigated probabilistic diffusion tractography in DYT1-negative patients with cervical dystonia and matched healthy control subjects, with the goal of showing that patients exhibit altered microstructure in the connectivity between the pallidum and brainstem. The brainstem regions investigated included nuclei that are known to exhibit strong connections with the cerebellum. We observed large clusters of tractography differences in patients relative to healthy controls, between the pallidum and the brainstem. Tractography was decreased in the left hemisphere and increased in the right hemisphere in patients, suggesting a potential basis for the left/right white matter asymmetry we previously observed in focal dystonia patients.

**Conclusions/Significance:**

These findings support the hypothesis that connections between the basal ganglia and brainstem play a role in the pathophysiology of dystonia.

## Introduction

In addition to the basal ganglia, the pontine brainstem [1], [2], [3], [4], and cerebellum [5], [6], [7], [8], [9], [10], [11], [12], [13], [14], [15], [16], [17], [18], [19], [20] have been implicated in dystonia by numerous studies. As a synthesis of this literature and a number of other observations in dystonia, we recently hypothesized that the pallidal output neurons exhibiting extensive collateralization to the brainstem [21], [22], [23] are the neurons gating the functional system that is affected in dystonia. While many pallidothalamic fibers collateralize to the pedunculopontine nucleus (PPN), a subset of them collateralize more extensively and project to both the PPN and the red nucleus (RN) [21]. More broadly, we proposed that this latter set of projections helps to coordinate a network of regions involved in the neural control of posture and stabilization [24], including both static and dynamic programs, and that these programs may be affected in a number of movement disorders. We hypothesized that the pedunculopontine nucleus (PPN) would be involved in maintenance of resting muscle tone and posture while the cerebellum, via connections with the red nucleus (RN), would encode balance- and gait-related postural control.

Our hypotheses were driven, in part, by a previous finding of abnormal white matter microstructure in focal dystonia patients in a region through which some of these collateralizing pallidal fibers project [25]. Two aspects of the 2006 diffusion tensor imaging (DTI) study [25] required replication and extension to address the following: (1) The finding was identified in a small subject population, (2) Further studies were required to identify the likelihood that the white matter asymmetry reflected changes to pallidal output fibers exhibiting extensive axon collateralization, including collateral projections to the brainstem. The current study addressed each of these issues.

Specifically, we evaluated brain microstructure in a larger cohort of cervical dystonia patients than in our previous DTI study, and used a combination of fractional anisotropy (FA), mean diffusivity (MD), and probabilistic diffusion tractography (hereafter referred to simply as “tractography”) to evaluate the integrity and density of pallidal projections to (or from) the brainstem, including those to the RN and PPN. While cerebellar tractography has been shown to be abnormal in genetic forms of dystonia [7], and microstructure in the putamen and pallidum has been shown to be abnormal in cervical dystonia [26], [27], tractography between the basal ganglia and the RN and PPN has not been evaluated in any form of dystonia. Thus, in addition to showing more evidence that there are abnormalities in the basal ganglia circuitry in dystonia patients, the current study aimed to add to the body of literature relating the brainstem and the cerebellum (which exhibits strong connections with the red nucleus) to dystonia, and to show evidence in humans with dystonia that communication between the basal ganglia and brainstem may be relevant to the disorder [6], [10].

## Methods

### Ethics Statement

All subjects included in this study signed written informed consent prior to participation in the study, and the study was approved by the Institutional Review Board of Massachusetts General Hospital (Partners Human Research Committee). All experiments were conducted in accordance with the principles of the Declaration of Helsinki.

To investigate the above questions, we used several approaches complementary to our previous region of interest (ROI) analysis in a larger cohort of cervical dystonia patients. This included a voxel-wise approach to measuring white matter FA and mean diffusivity (MD) in several *a priori* areas of evaluation (AOEs), and using probabilistic diffusion tractography (hereafter referred to as “tractography”) to test whether there were alterations in the microstructure of projections between either the pallidum or the ansa lenticularis (AL) and the brainstem/cerebellum. We investigated tractography from the entire pallidum (both the external pallidum [GPe] and internal pallidum [GPi]) in each hemisphere to capture all potentially altered pallidal output and/or input tracts. We separately investigated tractography from the anterior portion of the AL, since this is the portion of the tract where the fibers are most densely packed and therefore contains the lowest percentage of other tracts.

### Subjects

Twelve patients with primary focal cervical dystonia who were negative for the DYT1 mutation, and twelve healthy control subjects matched one-to-one for age (within three years), gender, and handedness (assessed by the Edinburgh Handedness Inventory [28]) participated in this study. Table 1 characterizes the demographics and clinical profiles for each patient, including duration of dystonia and past or present medication status. For those patients who were currently being treated with botulinum toxin (BTX) injections, scanning was conducted at the end of a treatment period (within the week before the next scheduled injections) so that symptoms were at their worst, and effects of botulinum toxin minimal. Four of the twelve patients had been included in our previous report of DTI abnormalities in focal dystonia [25] (hereafter referred to as “our previous DTI study”), in which we showed an abnormal hemispheric asymmetry in white matter medial to the pallidum in cervical and focal hand dystonia patients. This asymmetry was measured as a direct, within-subjects left/right comparison of FA medial to the pallidum using a region of interest analysis. This study also showed that the asymmetry normalized after treatment with BTX. The analyses conducted in the current study were completely distinct from those in the previous study, and expand our understanding of the findings in that study.

**Table 1:** Clinical characteristics of cervical dystonia patients.

patient #	age/handedness/gender	affected regions	side affected	scale scores	duration of dystonia	prior BTX injections?	neuroactive medications
1*	55/left/F	Neck, with	both, with left	BFM: 6; Tsui: 8	35 years	12 prior inj	eletriptan
	(ctrl:52/left/F)	dystonic	head tilt				
		tremor					
2*	54/right/M	Neck	both, with right	BFM: 6; Tsui: 10	19 years	4 prior inj	None
	(ctrl:56/right/M)		head tilt				
3*	35/right/F	Neck, trunk,	both, with left	BFM: 18; Tsui: 4	13 years	10 prior inj	None
	(ctrl:34/right/F)	craniofacial	head tilt, right				
			rotation, right				
			craniofacial				
4*	57/right/F	Neck, with	both, with left	BFM: 4; Tsui: 7	41 years	11 prior inj	None
	(ctrl:59/right/F)	dystonic	head tilt,				
		tremor	retrocollis				
5	59/R/M	Neck	right tilt	BFM: 4; Tsui: 8; TW: 22	20 years	25 prior inj	None
	(ctrl: 60/right/M)						
6	46/R/M	Neck	right tilt, shoulder	BFM: 4; Tsui: 4; TW: 17	7 years	9 prior inj	Tramadol
	(ctrl: 45/right/M)		elev				
7	58/R/F	Neck	right tilt	BFM: 1; Tsui: 3; TW: 9	3 months	no	citalopram for minor
	(ctrl: 57/right/F)						depression
8	46/R/M	Neck	both, with left	BFM: 6; Tsui: 9; TW: 23	7 years	1 inj four	2002 diazepam, none
	(ctrl: 46/right/M)		rotation, right tilt,			years ago	currently
			anterocollis				
9	37/R/M	Neck	both, with left tilt,	BFM: 2; Tsui: 3; TW: 19	3–4 years	1 prior inj	clonazepam in Jan08,
	(ctrl: 37/right/M)		right lateral shift				but none at time of
							scan
10	38/R/F	Neck	both, with right	BFM: 4; Tsui: 6; TW: 21	8 or 9	2 prior inj	diazepam as needed,
	(ctrl: 41/right/F)		rotation, left tilt		years		last dose 3–4 wks
							before scan
11	50/R/F	Neck	right rotation	BFM: 8; Tsui: 6; TW: 17	5 months	no	0.5 mg clonazepam
	(ctrl: 49/right/F)						
12	55/R/M	Neck	left rotation	BFM: 2; Tsui: 3; TW: 15	5–6 years	no	None
	(ctrl: 54/right/M)						

* subjects included in 2006 study using different analyses.

BFM: Burke Fahn Marsden dystonia rating scale.

Tsui: Tsui rating scale for cervical dystonia.

TW: Toronto Western Spasmodic Torticollis Rating Scale (TWSTRS) for cervical dyston.

### Diffusion tensor imaging (DTI) imaging protocol

All subjects were scanned using a standard high resolution DTI protocol. The eight patients not included in the 2006 study and their matched control subjects were scanned on a 3.0 Tesla Siemens Tim Trio magnet system (Siemens AG, Medical Solutions, Erlangen, Germany); the four patients who were studied in the 2006 publication had been scanned on a Siemens 3.0 Tesla Allegra Magnet System. One-to-one matched patient and control pairs were always scanned in the same magnet, and each subject with multiple scanning sessions was also always scanned in the same magnet. Thus, the change in magnet was not a factor in any of our group or treatment comparisons. Images were acquired using a high-resolution whole brain DTI sequence with the following sequence parameters: repetition time (TR) = 24 s; echo time (TE) = 81 ms; slice thickness = 2 mm isotropic, 60 slices total, acquisition matrix 128×128 [256×256 mm field of view (FOV)], six averages, 60 noncolinear directions, with b-value = 700 s/mm^2^, and one image with b-value = 0 s/mm^2^. DTI scans in each subject were acquired using auto-align software [29] to normalize brain image slice orientation between participants.

FA and tractography do not convey any information about absolute direction of water diffusion. These measures evaluate only information about number of directions of diffusion (FA) and relative directions in one voxel relative to adjacent voxels (tractography) [30]. Nevertheless, in our previous DTI study we used rigorous procedures to be certain that possible deviations of patient head position did not influence DTI measures, and it was determined that head position had no influence on these measures. Two separate precautions were taken. The first was that the use of autoalign software [29] ensured that slices were acquired in the same orientation across subjects and/or scanning sessions within a subject (aligned with the AC/PC line, independent of head position). With this software, images are acquired relative to the head itself, not absolute to position of the head in the scanner. The second precaution was to match control subjects' head positions across the two sessions in which they were scanned, to the head positions of the patients across sessions; that is, control subjects' heads were tilted or turned such that the angle matched that of the patient with which they were matched. We used these measures to determine (1) if this led to any asymmetries in white matter measures and (2) if controls showed changes in left/right measures across scanning sessions with different head positions. Our results confirmed that head position in control subjects did not lead to any left/right white matter asymmetries, and that there were no changes in left/right hemispheric differences across scanning sessions/head positions in these control subjects. Given the above points, we can be confident that head position in the scanner did not influence the DTI results reported here. As an additional precaution in the current study, we also assessed whether there was any effect of group on amount of head movement during scanning. We did this by using a two-way ANOVA to assess the effects of group and of the interaction of group by direction of movement. We used the values from the output of the preprocessing motion correction step, which includes information for rotation around and translation within the x, y, and z planes. We found no effect of group, or of interaction between group and direction of movement (F = 0.7 for group effect (p = 0.41), F = 0.78 for group×direction interaction (p = 0.57).

### Data analysis: General

All image analysis was performed using tools from The Oxford Centre for Functional Magnetic Resonance Imaging of the Brain (FMRIB) software (www.fmrib.ox.ac.uk/fsl) version 4.1.4, using standard parameters. Specifically, we used FMRIB's Diffusion Toolbox (FDT v2.0).

#### Preprocessing

The initial data preprocessing for each subject included radiological orientation, removal of non-brain tissue (BET), and correction for head motion and eddy current distortions. DTIFIT reconstruction of diffusion tensors at each voxel was applied to create 3D images at the same matrix size and resolution as the original diffusion images, including FA and MD maps for each subject.

#### Registration procedures

FA maps were registered into MNI standard space using the FSL non-linear transform (i.e. FNIRT), registering to the FSL DTI template FA map (FMRIB58_FA_1mm_brain). Other maps used in the analyses (MD, tractography), and seed regions for tractography, were registered using the same transform matrix used for registering FA maps.

### Data analysis: FA and MD map contrasts

We used the FSL program *Randomise* to compute a voxel-wise t test for the contrast of FA and MD maps for the 12 patients versus 12 controls (group contrast). This software is the standard FSL software used for DTI group contrast analyses. Randomise uses a permutation test to evaluate differences between groups, and is thus particularly well suited to populations which we cannot assume are normally distributed (http://www.fmrib.ox.ac.uk/fsl/randomise/index.html). In these contrasts, we used variance smoothing to account for (1) variance in spatial co-localization of small tracts in standardized space and (2) greater expected variance of signal amplitude across patients than across controls in DTI measures in this region, based on our previous findings [25]. We chose not to use the FSL tbss skeletonized analysis given the small, subcortical tracts we were evaluating, particularly when these tracts were known to cross the main direction of the skeleton, rather than run along the skeleton. The overlap of each of our segmented regions with the skeleton was only a few voxels and thus this approach did not provide enough “signal” for our purposes.

#### Significance thresholds for FA and MD contrasts

Significant differences between groups were determined based on meeting both of the following criteria: (1) a significance threshold for peak t values, corrected for multiple comparisons using a bonferroni correction, and (2) a cluster threshold. The procedures we used to correct for multiple comparisons are standard for imaging studies [31] and, in fact, exceed several other standard correction methods in their level of stringency [7], [32].

#### (1) Bonferroni correction

Our t threshold was corrected for multiple comparisons using a bonferroni correction, based on the number of voxels included in our search volume (i.e. *a priori* AOEs) divided by the number of voxels in the cluster threshold (p = 0.05/[searchvol/clustervol]). *A priori* AOEs included the ansa lenticularis (AL) (for the pallidal seed region only), the substantia nigra (SN), the red nucleus (RN), the superior cerebellar peduncle (SCP), and the pedunculopontine nucleus (PPN); these regions were segmented by an anatomist (N.M.) using landmark-based, atlas-guided definitions of the regions. Descriptions of these segmentations and illustrative examples of each one can be found in Methods S1 and Figure S1. Based on the 8,007 1 mm isotropic voxels across these AOEs (multiplied by two to account for both FA and MD contrasts) and a cluster threshold of 72 (described below), the corrected t threshold for statistical significance with df = 22 was p<0.00022, or t<4.42 (correction of p<0.05 for 8,007×2 voxels in search region divided by the 72 cluster threshold, using a two-tailed test).

#### (2) Cluster threshold

We required peak t values to be part of a minimum size cluster of 72 contiguous voxels at p<0.05 uncorrected (t<2.07 with df = 22, two-tailed) to be considered for significance. This cluster threshold was based on a requirement of nine, 2 mm isotropic contiguous voxels in *a priori* AOEs in our recent DTI study [33], a volume which exceeded cluster thresholds previously used with fMRI [34], [35] and cortical thickness [36]. Given that the current voxelwise analysis was done using images registered to a 1 mm isotropic template, we therefore required 9×8 or 72 voxels to match the same volume as the cluster as our previous study.

### Data analysis: Probabilistic diffusion tractography

Tractography maps were created for each subject using the FSL *probtrackx* program; maps were created separately for the left and right seed regions. *Probtrackx* is part of the FSL diffusion toolkit (FDT V 2.0) that generates connectivity distributions from a seed point or region. To generate the files needed for *probtrackx*, *bedpostx* was run on preprocessed DTI data in each subject's native space. *Bedpostx* is also a part of FDT v2.0 that builds distributions on diffusion parameters at each voxel and determines the number of crossing fibers per voxel. We next specified our seed regions of interest, the left pallidum, right pallidum, left AL, and right AL, using the methods described in the paragraphs below. *Probtrackx* was then set to calculate 5000 samples (default value) from each seed region and a voxelwise map was constructed where each voxel had a value representing the total number of samples received from the seed region. We used a curvature threshold of 0.2 (default value) which represents a maximum allowable angle of ±80 degrees. The resulting maps were then registered to MNI standard space according to the methods described below.

#### Creating the seed regions for each subject

The pallidal seed region was created using the following procedure: We began by segmenting the left and right pallidum in MNI space following the Center for Morphometric Analysis MRI-based anatomical methodology as described in Filipek et al (1994). For the AL seed region we used the segmentation of the anterior AL used as one of our *a priori* AOEs in the FA analysis (see above and Methods S1, Figure S1A). Segmentations, which were in standard MNI space, were projected back into native DTI space to compute tractography maps, since tractography itself is always run in diffusion (i.e. native) space (http://www.fmrib.ox.ac.uk/fsl/fdt/fdt_probtrackx.html). To do this, we used the inverse transforms of the registration matrices of FA maps to standard space.

We also verified, as a negative control, that there were not systematic differences in the volume of the pallidum and AL between patients and controls. There were no significant differences in volume in these structures/regions (left pallidum, right pallidum, left AL, right AL) in patients, relative to controls (p values for these comparisons ranged from 0.35 to 0.76, uncorrected).

#### Contrasts for tractography

Once tractography maps were in MNI space, voxel-wise contrasts evaluating the 12 patients versus 12 controls were run for the maps generated from each seed region using the FSL program, *Randomise*, with variance smoothing, as for FA contrasts.

#### Significance thresholds for tractography contrasts

No previous standardized criteria have been established to evaluate significance thresholds for voxel-wise contrasts of tractography maps. In this study we developed what we believe to be very conservative methods for establishing statistical significance for these contrasts, based on meeting both (1) bonferroni corrections for t thresholding and (2) cluster thresholding, similar to the FA and MD map contrasts. In imaging studies, the bonferroni/cluster correction approach is generally considered the most stringent method of correction as it leads to higher thresholds for significance than other approaches [32], such as false discovery rate (FDR) [7]. At this stage of our research, we preferred to err on the side of risking potential type II errors, rather than type I errors, particularly since our study was guided by strong hypotheses.

#### (1) Bonferroni corrections

Our *a priori* search volume for the tractography analysis included all white matter regions and nuclei that potentially contain projections between the pallidum and the pons and cerebellum. In order to correct for multiple comparisons we determined the number of voxels in our search volume by segmenting the brain from the level of the basal ganglia downwards, excluding cerebellar cortex (see Methods S1, Figures S2, S3, S4). We did not include regions anterior or posterior to the basal ganglia, or in the temporal lobe, because the tracts of interest do not project to these regions. The total voxel count in the segmentation of our search volume for both hemispheres was 64,732. Since tractography does not perform well across decussations [7], we limited our search for each seed region contrast to the hemisphere in which the seed region was located, resulting in a search volume of 32,366 per contrast. Based on this search volume and our cluster threshold of 291 (see below), for each patient/control contrast, a peak t value greater than 4.70 was required for group differences that met the cluster threshold to reach statistical significance (p<0.00011 with df = 22 after correction of p<0.05 for 32,366×4 voxels in search region divided by the 291 cluster threshold, using a two-tailed test).

#### (2) Cluster threshold

Since we were evaluating long white matter tracts rather than distinct foci within nuclei, our search volume spanned large numbers of voxels whose values we assumed would not be entirely independent of one another. Consequently, we increased the size of the cluster threshold relative to the FA contrast, in proportion to the increase in the search volume from the FA contrast. The cluster threshold for each contrast was thus calculated as: 72[FAclustervol]x(32,366[tractogsearchvol]/8,007[FAsearchvol]) = 291 voxels. Thus, we required a minimum of 291 contiguous voxels at p<0.05 uncorrected (two-tailed) *within the segmented* a priori *search volume* in order for a region to be considered for significance. For df = 22 in the patient versus control contrasts, p<0.05 corresponded to a t value of 2.07.

Although only one peak t value was required to meet the bonferroni correction in each difference cluster, clusters passed through and between multiple *a priori* AOEs used in the FA map contrast. To indicate the distribution of t values across the cluster, tables report peak t values in each cluster for all *a priori* AOEs overlapped by the cluster if they reached a t value of 3.5 or greater; statistical significance is indicated for t values that met the t>4.12 threshold. If the peak t value in a region was below 3.5, the region was listed in the tables, but without a t value. However, since we were evaluating white matter, differences were not necessarily expected to be strongest within nuclei themselves, but in the white matter projecting to these nuclei; if the overall peak for a cluster was in white matter between *a priori* AOEs, this was indicated along with the AOEs it ran between.

## Results

### FA and MD analyses

#### Voxel-wise contrasts of FA and MD maps for patients versus controls

**Figure 1:**
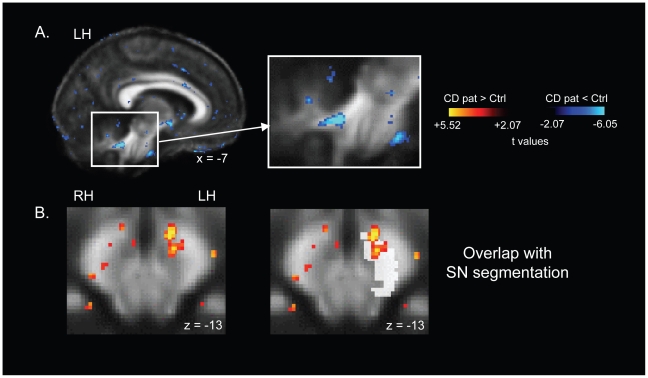
Significant FA differences in cervical dystonia patients relative to control subjects. (A) Reduced FA in the left cerebellar white matter in patients. (B) Increased FA adjacent to and overlapping with the left substantia nigra in patients. MNI talairach coordinates are indicated for each image. t maps are superimposed on the average FA map for all 24 subjects in the study, and are thresholded at t = +/−2.5 here for illustrative purposes. The color bar indicates the range of t values in this figure for FA contrasts, from +/−2.5 to the peak positive and negative t values for this contrast. Warm tones (red, orange, yellow) indicate regions in which cervical dystonia patients exhibited elevated FA relative to control subjects. Cool tones (blues) indicate regions in which cervical dystonia patients exhibited reduced FA relative to control subjects. LH: left hemisphere; RH: right hemisphere.

Within our *a priori* AOEs for the voxel-wise contrast, cervical dystonia patients exhibited reduced FA in white matter overlying the left SCP and a trend toward reduced FA in the right SCP, at the entry to the cerebellum (Table 2; Figure 1A). There was also a trend toward significantly reduced FA overlying the left ansa lenticularis (AL) in patients. Finally, FA was elevated anterior to and within the left substantia nigra (SN) in patients (Figure 1B, left column). There were no group differences in MD in any of our *a priori* AOEs.

**Table 2:** Group differences for voxel-wise FA contrast (12 cervical dystonia patients versus 12 matched controls).

Region	MNI coordinates at peak difference	t values at peak difference	Cluster size(# voxels)
L. SCP	−7 −43 −28	−6.05*	214
R. SCP	5 −48 −27	−4.23†	271
L. AL	−7 2 −8	−3.96†	40
L. SN/SN wm	−7 −11 −13	+5.52*	91

* Met significance criteria (p<0.00022, [p<0.05, corrected, or t = 4.42] for t values, and 72 voxels for cluster threshold).

† p value was within an order of magnitude of the corrected threshold (a trend).

Positive t values indicate FA values were elevated in cervical dystonia patients relative to control subjects; negative t values indicate FA values were reduced in cervical dystonia patients relative to control subjects. L = left hemisphere; R = right hemisphere; wm = white matter.

### Probabilistic diffusion tractography analyses

#### Characterization of tractography maps

**Figure 2:**
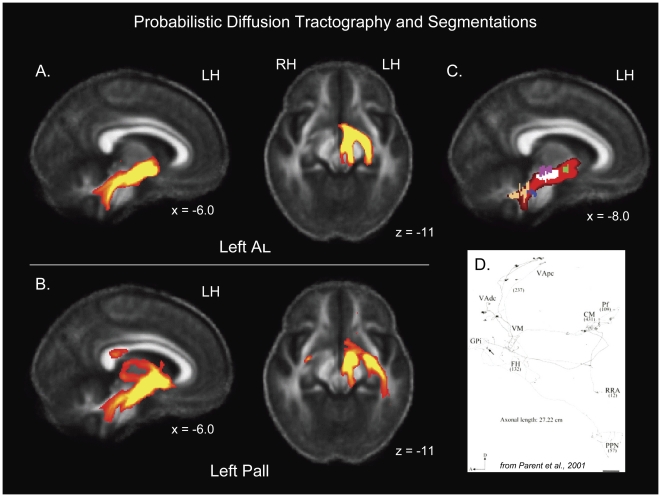
Probabilistic diffusion tractography and overlap with *a priori* areas of evaluation (AOEs) used for the FA and MD contrasts. (A) Examples of tractography from the left ansa lenticularis (AL) seed region, averaged across control subjects (before contrasts were conducted), thresholded at 500 (samples). Note that tractography bifurcated at the level of the substantia nigra (see axial image). (B) Examples of tractography from the left pallidal seed region, averaged across control subjects (before contrasts were conducted), thresholded at 500. Like AL tractography, pallidal tractography bifurcated at the level of the substantia nigra (see axial image). There were also two projections to the thalamus, one superior and one more ventral (see sagittal image). (C) Intersection of tractography with segmentations of our *a priori* AOEs, including (in descending order) the ansa lenticularis (green), the substantia nigra (white), the red nucleus (pink), the pedunculopontine nucleus (blue), and the superior cerebellar peduncle (peach). Tractography maps are superimposed on the average FA map for all 24 subjects in the study. (D) Example of type Ib GPi neuron from [21], showing extensive arborization, including to the RN and PPN (reprinted with permission from The Journal of Comparative Neurology). MNI talairach coordinates are indicated for all images. LH: left hemisphere; RH: right hemisphere.

Tractography overall looked qualitatively similar between patients and controls (Figure 2A,B), as well as across hemispheres within each group. When thresholded to limit viewing of connections at a probability of 500 and above (i.e. an arbitrary threshold set to illustrate the regions with the strongest likelihood of connection to the seed region), the main regions of projection from the pallidum and the AL seed regions were (1) to the thalamus and (2) to the brainstem, running through the SN and the edge of the RN, and then branching to (a) the cerebellum and (b) the PPN (Figure 2, sagittal images). At the level of the SN, there was a bifurcation of tractography, with one branch running through the medial SN, and the other through to the lateral SN (Figure 2, axial images). The branch of tractography to the cerebellum may have reflected cerebellothalamic fibers that co-mingled with pallidal fibers projecting down the brainstem, since at least some of these fibers collateralize within Forel's field H. Alternatively, since tractography does not “see” synapses or detect the direction of tracts, this branch may have reflected cerebellar projections to the RN which were contiguous with ipsilateral projections between the RN and pallidum or AL, or an intrahemispheric relay from the cerebellar dentate nucleus to the pons to the STN [37]. Figure 2C shows where tractography was observed relative to our segmented *a priori* AOEs used in the FA and MD contrasts.

#### Voxel-wise contrasts of tractography maps for patients versus controls

**Figure 3:**
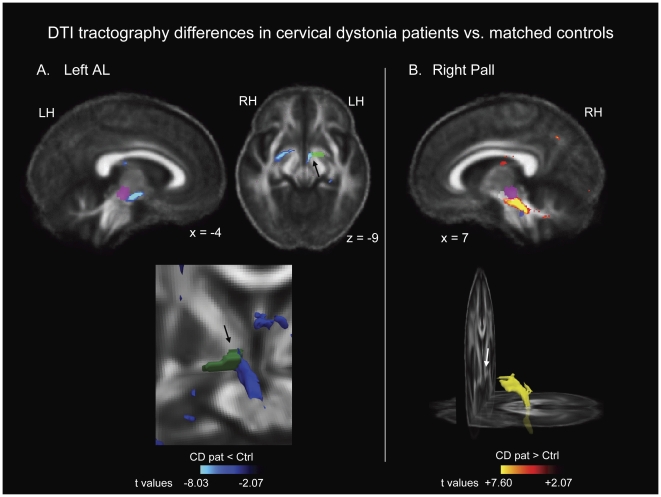
Regions of probabilistic diffusion tractography that were significantly different between cervical dystonia patients and controls. (A) shows reduced tractography from the left AL seed region, and (B) shows elevated tractography from the right pallidal seed region. Arrows point to the region through which we drew ROIs in our previous DTI study, which includes AL fibers. *A priori* segmented regions are shown as reference points: Pink = red nucleus; White = substantia nigra; Blue = pedunculopontine nucleus. MNI talairach coordinates are indicated for each two dimensional image. Lower images in each panel show three dimensional rendering of clusters; the image for the left AL includes the AL seed region in green, and the patient/control difference is shown in blue. t maps and three dimensional clusters are superimposed on the average FA map for all 24 subjects in the study for anatomical reference, and are thresholded at t = +/−2.07 (the threshold used to identify difference clusters, p<0.05, uncorrected, for df = 22). The color bars indicate the range of t values in each panel, from +/−2.07 to the peak t value for each contrast. Warm tones (red, orange, yellow) indicate regions in which cervical dystonia patients exhibited elevated tractography relative to control subjects. Cool tones (blues) indicate regions in which cervical dystonia patients exhibited reduced tractography relative to control subjects. Three dimensional images are shown in mono-color rather than graded/multi-color to illustrate location rather than significance. LH: left hemisphere; RH: right hemisphere.

A voxel-wise contrast of tractography differences between patients and controls indicated that patients exhibited reduced probability of connectivity between the left AL and left brainstem, running through regions medial and inferior to the AL, to the SN and RN (Table 3; Figure 3A). The difference in this cluster was greatest in white matter between the AL and the RN/SN. Patients also exhibited increased probability of connectivity between the right pallidum and right brainstem, running through the SN, RN, PPN, SCP region within the pons, and continuing down the brainstem, but not into the cerebellum (Figure 3B); this cluster did not run through the AL region. The difference in this cluster was greatest in the white matter running between the SN/RN and the PPN, and in the adjacent SCP region. Although a separate cluster meeting the cluster threshold was observed in cerebellar white matter, it did not reach the t threshold for significance. Difference clusters in both hemispheres fell entirely within the *a priori* brainstem search volume and/or the seed regions.

**Table 3:** Group differences for voxel-wise probabilistic diffusion tractography contrast (12 cervical dystonia patients versus 12 matched controls).

Region	MNI coordinates at peak difference	t values at peak difference	Cluster size(# voxels)
**Left AL seed region**			
*L. Pallidum/Brainstem, including:*			
wm between AL and RN/SN	−5 −8 −11	−8.03*	442*
SN	−5 −10 −11	−6.87*	
RN	−4 −16 −13	−3.76	
**Right Pallidum seed region**			
*R. Brainstem, including:*			
SN	8 −26 −16	5.04*	1241*
RN			
wm between RN/SN and PPN	7 −29 −21	7.21*	
PPN	6 −32 −25	6.07*	
SCP	7 −30 −21	7.60*	
*Cerebellar cortex wm*	29 −57 −42	4.18^†^	488*

* Met significance criteria (p<0.00011 [p<0.05 corrected, or t = 4.70] for t values, and 291 voxels for cluster threshold).

Negative t values indicate that tractography measures were reduced in cervical dystonia patients relative to control subjects. Positive t values indicate that tractography measures were elevated in cervical dystonia patients relative to control subjects. Note that all regions included in the cluster are reported; however, only one peak within the cluster was required to reach statistical significance (t>4.12). t values are reported for all regions exhibiting peaks of 3.5 or greater. L = left hemisphere; R = right hemisphere; wm = white matter.

#### Location of patient/control FA differences relative to patient/control tractography differences

The left hemisphere cluster of decreased tractography in patients relative to controls ran adjacent to, and overlapped the ventral edge of the cluster of elevated FA in the SN in patients. The clusters of reduced FA overlying the left and right SCP in patients did not overlap with the clusters of patient/control tractography differences.

## Discussion

We observed FA and tractography differences in cervical dystonia patients that were consistent with our hypothesis that pallidal output fibers to the brainstem are altered in some way in this disorder [24], [25]. There was evidence for altered connectivity between the left AL and ipsilateral brainstem, and between the right pallidum and ipsilateral brainstem in cervical dystonia patients. The left and right hemispheres were affected in opposite directions, with decreases and increases in probabilistic tractography, respectively. Moreover, only the left hemisphere showed differences in and immediately adjacent to the region where we previously observed an FA asymmetry medial to the pallidum in focal dystonia patients ([25]; hereafter referred to as “our previous DTI study”).

### The anatomy of FA and tractography differences in cervical dystonia before treatment

Although tractography does not have the specificity to define exactly which tracts it has detected, we can evaluate our findings in the context of existing hypotheses, as well as use clues from the combination of FA and tractography data to make potential inferences about anatomy and etiology as a hypothesis-generating step. One of the main hypotheses in our conceptual model for dystonia [24] was that pallidal output fibers that collateralize to both the RN and the PPN (Type Ib fibers; [21]) are the neurons gating the functional system that is affected in dystonia. Specifically, we suggested that these fibers are positioned to coordinate a number of spatially distinct regions controlling different components of posture and stabilization, including serving as a “toggle” between rest and movement-related postural control programs. According to this model, different forms of dystonia reflect excessive function in different subcomponents of posture and stabilization. Such excessive function might result either from faulty gating of postural programs, or from malfunction of the downstream programs themselves. The relative density of Type Ib neurons relative to other neurons, the amount of collateralization of a given fiber, or the microstructural properties of the fibers themselves would influence both FA and tractography because of their reduced coherence. Although we cannot be certain the Type Ib fibers were the source of altered tractography in our current study, rather than other pallidal or striatal afferents or efferents, the abnormal tractography we observed spanning from the AL and pallidum to both the RN and PPN supports the potential importance of these fibers to dystonia. Figure S5 schematizes ways in which these fibers might affect FA and tractography. Note that these fibers project through multiple pallidal output tracts, and are not limited to projections through the AL.

The opposite directions of tractography differences in patients in the left versus right hemispheres identified a potential basis for the hemispheric asymmetry observed in our previous study; in addition, only in the left hemisphere were tractography differences in patients observed in and adjacent to the region where we previously drew our ROIs. Because only the left hemisphere differences were detected using the AL seed region, this suggests that the tract(s) affected in the left hemisphere were more likely to have corresponded to fibers in the AL projection from the GPi, whereas the right hemisphere differences observed from the pallidal seed region may have been in fibers leaving the GPi through a different tract, such as the lenticular fasciculus, or fibers going to/from the STN.

Difference clusters in the analyses here sometimes extended below the PPN and, interestingly, they appeared to extend into the region containing the vestibular nuclei. Although these nuclei were not part of our *a priori* hypothesized areas of interest in this study, the vestibular nuclei are known to work with the cerebellum to maintain equilibrium [38]. The fibers traced in the study by Parent and colleagues [21] similarly continued below the PPN. Thus, our findings in this region suggest the vestibular nuclei should be included in our *a priori* areas of interest in future studies.

Because we did not conduct tractography from the SN, it cannot here be determined what the relationship was between our tractography findings, which passed through the SN, and increased FA in the SN; the small amount of overlap of these two clusters at the edge suggests the possibility that they are related in some way. Interestingly, we recently observed elevated FA in the SN in a putative “subtype” of patients with major depressive disorder (MDD) [33]. Given the frequent comorbidity of dystonia and MDD [39], [40], [41], [42], it will be important to further pursue studies of the SN in both disorders, particularly in post mortem tissue.

#### Discerning basal ganglia fibers from other pathways in the AL region

In our previous study we hypothesized that the hemispheric asymmetries medial to the pallidum reflected abnormalities in pallidal output fibers. However, we acknowledged that, using FA measures, we could not rule out non-basal ganglia fibers in this region, including internal capsule fibers, amygdalofugal fibers, optic tract fibers, and anterior commissure fibers. In the current study, we used two approaches to provide additional evidence that our findings reflected differences in basal ganglia fibers. The first approach was using the pallidum itself as a seed region. The second was to verify that difference clusters we observed did not project to the other regions mentioned above, including sensorimotor cortex, the medial temporal lobe, visual cortex, or anterior to the AL toward the midline (where the anterior commissure projects); in no case were these other projections observed. In addition, none of the other candidate fibers listed above are known to project to the brainstem, with the exception of the internal capsule. The current findings therefore suggest that our previous findings are likely to have reflected abnormalities in basal ganglia input or output fibers, although we cannot rule out that abnormalities in other tracts may have also contributed to the previous FA findings.

### Etiology of altered connectivity

Observations of decreased tractography are generally interpreted as evidence for reductions in fiber number, integrity, coherence, or myelination in the affected pathways and, conversely, observations of increased tractography are generally interpreted as increases in one or more of these features. Given that DTI evaluates water diffusion, there could potentially be many alternate etiologies for our findings of abnormalities in patients, such as altered fast axonal transport [43] or glial density/structure around axons.

The observation of reduced tractography from the AL region in patients is consistent with our previous observation that left hemisphere FA in the AL region was lower than right hemisphere FA in this region in focal dystonia patients. Given our hypothesis that the pallidofugal fibers exhibiting the most extensive collateralization (type Ib, [21]) were more abundant in this region in patients in our previous DTI study, it is possible that more of these fibers and thus a reduced coherence of fibers in this region leads to reductions in both FA and apparent tractography. Figure S5 illustrates the potential opposing effects of coherence versus fiber number when there are collateralizing fibers present. Alternatively, the reductions in these measures might reflect fewer fibers or damaged fibers (either permanent damage, or reversible damage associated with excessive function, as observed in animal models of epilepsy [44]) in this region.

### Functional significance of hemispheric differences in dystonia

Our previous DTI study identified a left/right asymmetry in white matter medial to the pallidum in focal dystonia patients. The tractography findings here suggest it is not necessarily the left/right asymmetry in and of itself that is critical to the expression of dystonia, but that this was a functional or structural biomarker that led us to identify regions that were likely to show differences in tractography in dystonia patients. However, given that the direction of tractography results across hemispheres in the current study is consistent with the direction of the FA asymmetry observed previously (left hemisphere reduction and right hemisphere elevation), this leads to the question of why there appears to be a dominant direction of effect in a group of subjects who showed a fairly equal distribution of symptoms on the left versus right side of the body (see Table 1). We cannot yet be completely certain of the answer to this, but given that there is normally laterality/hemispheric dominance in motor control (e.g. handedness; all but one patient in this study was right-handed) [45], it would not be surprising if there were also hemispheric differences in the proportions and anatomy of pallidal output tracts in controls, which may be amplified, and thus more easily detected, in patients (e.g., suggesting that the scenario in Figure S5B may be more likely than the one in Figure S5A). Note that our findings here from the group contrast reflect the dominant group effect, which does not mean that all individuals necessarily show laterality in the same direction; furthermore, we did not directly assess within-subject asymmetry in this study as we did in the previous study.

Normal laterality in postural control systems is likely to be present in healthy subjects as a result of different demands on posture on the left versus the right side of the body. For example, one would expect relatively more demand on local stabilization programs on the dominant side of the body to control skilled movements such as writing and reaching, and relatively more demand on balance-related function on the opposite side of the body as postural compensation for extension of the opposite hand or arm. Indeed, many cervical dystonia patients show a mixture of static and dynamic symptoms that may reflect excessive function of these two different types of (asymmetrically applied) postural components. In our conceptual model we also propose that static and dynamic symptoms could potentially reflect elevated versus reduced function, respectively, of the pallidal output neurons. If density or microstructural features of these neurons show any correspondence to levels of function, this suggests a way in which structural asymmetries in dystonia might potentially correspond to functional asymmetries.

It cannot be determined at this time whether the tractography findings here correspond to cause or effect of the dystonia; nor can it be determined whether they reflect congenital/developmental anatomy versus plastic changes in the brain. Given the abundance of data regarding the role of plasticity in both symptom induction [46], [47], [48], [49] and response to treatment [50], [51], [52], [53] in dystonia, it will be important to address this question in the future.

### Summary

In this study, we have shown additional evidence for altered microstructure of basal ganglia output fibers in focal dystonia patients, and shown that the differences extended to the brainstem. These findings are consistent with our hypothesis that GPi fibers exhibiting collateralization to the brainstem are altered in dystonia. While we cannot be completely certain which pallidal afferents or efferents were affected, this is the first demonstration of altered connections between the pallidum and the brainstem in dystonia, and is considered part of a hypothesis-generating step to be tested in animal models of dystonia. Future tractography studies will aim to address whether individual differences in tractography predict individual differences in the clinical presentation of dystonia.

## Supporting Information

Methods S1 **This supporting information section describes the segmentation procedures used to define the search volumes for patient versus control contrast analyses.** The first section describes the segmentation of *a priori* areas of evaluation (AOEs) for FA and MD contrasts, and the second section describes segmentation of the *a priori* search volume for the tractography contrast.
(DOC)

Figure S1 **Examples of segmentations for each** ***a priori*** **area of evaluation (AOE) used, collectively, as the search volume in the FA and MD contrasts.** Segmentations are shown in white, superimposed on the average FA brain for all 24 subjects in the study. MNI coordinates are indicated for each image. (A) Segmentation of the ansa lenticularis (AL) (B) Segmentation of the substantia nigra (SN) (C) Segmentation of the red nucleus (D) Segmentation of the pedunculopontine nucleus (E) Segmentation of the superior cerebellar peduncle.
(TIF)

Figure S2 **Examples of the segmentation used as the** ***a priori*** **search volume in the tractography contrasts, shown from a sagittal view.** MNI talairach coordinates are indicated for each image. LH: left hemisphere.
(TIF)

Figure S3 **Examples of the segmentation used as the** ***a priori*** **search volume in the tractography contrasts, shown from a coronal view.** MNI talairach coordinates are indicated for each image. LH: left hemisphere; RH: right hemisphere.
(TIF)

Figure S4 **Examples of the segmentation used as the** ***a priori*** **search volume in the tractography contrasts, shown from an axial view.** MNI talairach coordinates are indicated for each image. LH: left hemisphere; RH: right hemisphere.
(TIF)

Figure S5 **This figure suggests some theoretical ways in which two primary types of motor neurons projecting from the pallidum** [**21**] **might influence diffusion tensor imaging (DTI) measures.** The primary issue at hand is that one fiber type, Type Ib, exhibits much more extensive collateralization than the other (Type Ia), even within the region immediately medial to the pallidum, and that collateralization reduces axon coherence. Given that axon coherence is a major component influencing fractional anisotropy (FA), if the relative proportion of these fibers is increased, FA will decrease. Because anisotropy influences probabilistic tractography, tractography will also be reduced if the relative proportion of these fibers is increased, perhaps even if there are more fibers projecting through the region. (A) Shows the effects that collateralization will have on FA, with examples of how these effects might be altered if the relative numbers of fibers in the left versus right hemispheres (1) or the amount of collateralization from existing fibers (2) increases or decreases in dystonia. While (1) is more likely to develop from asymmetric loss of neurons (less likely in primary dystonia), it is not out of the question that (2) could take place as a microstructural response to changes in function, given that terminal arborization has been shown to take place over weeks [54]. (B) Suggests the possibility that healthy individuals exhibit asymmetries in the left/right proportions of the Type Ib fibers relating to normal motor laterality, which only become detectable with DTI when there is a relative increase in the number of these fibers in proportion to other fibers in this region that collateralize less (e.g. Type Ia fibers), or not at all (descending internal capsule fibers).
(TIF)

## References

[1] Loher TJ, Krauss JK (2009). Dystonia associated with pontomesencephalic lesions.. Mov Disord.

[2] Tan EK, Chan LL, Auchus AP (2005). Hemidystonia precipitated by acute pontine infarct.. J Neurol Sci.

[3] McNaught KS, Kapustin A, Jackson T, Jengelley TA, Jnobaptiste R (2004). Brainstem pathology in DYT1 primary torsion dystonia.. Ann Neurol.

[4] Wu CL, Lu CS (1992). Delayed-onset dystonia following recovery from central pontine myelinolysis.. J Formos Med Assoc.

[5] Carbon M, Argyelan M, Habeck C, Ghilardi MF, Fitzpatrick T (2010). Increased sensorimotor network activity in DYT1 dystonia: a functional imaging study.. Brain.

[6] Bostan AC, Strick PL (2010). The cerebellum and basal ganglia are interconnected.. Neuropsychol Rev.

[7] Argyelan M, Carbon M, Niethammer M, Ulug AM, Voss HU (2009). Cerebellothalamocortical connectivity regulates penetrance in dystonia.. J Neurosci.

[8] Teo JT, van de Warrenburg BP, Schneider SA, Rothwell JC, Bhatia KP (2009). Neurophysiological evidence for cerebellar dysfunction in primary focal dystonia.. J Neurol Neurosurg Psychiatry.

[9] Brighina F, Romano M, Giglia G, Saia V, Puma A (2009). Effects of cerebellar TMS on motor cortex of patients with focal dystonia: a preliminary report.. Exp Brain Res.

[10] Neychev VK, Fan X, Mitev VI, Hess EJ, Jinnah HA (2008). The basal ganglia and cerebellum interact in the expression of dystonic movement.. Brain.

[11] Zadro I, Brinar VV, Barun B, Ozretic D, Habek M (2008). Cervical dystonia due to cerebellar stroke.. Mov Disord.

[12] Carbon M, Ghilardi MF, Argyelan M, Dhawan V, Bressman SB (2008). Increased cerebellar activation during sequence learning in DYT1 carriers: an equiperformance study.. Brain.

[13] Delmaire C, Vidailhet M, Elbaz A, Bourdain F, Bleton JP (2007). Structural abnormalities in the cerebellum and sensorimotor circuit in writer's cramp.. Neurology.

[14] Le Ber I, Clot F, Vercueil L, Camuzat A, Viemont M (2006). Predominant dystonia with marked cerebellar atrophy: a rare phenotype in familial dystonia.. Neurology.

[15] Lehericy S, Gerardin E, Poline JB, Meunier S, Van de Moortele PF (2004). Motor execution and imagination networks in post-stroke dystonia.. Neuroreport.

[16] Ghilardi MF, Carbon M, Silvestri G, Dhawan V, Tagliati M (2003). Impaired sequence learning in carriers of the DYT1 dystonia mutation.. Ann Neurol.

[17] LeDoux MS, Brady KA (2003). Secondary cervical dystonia associated with structural lesions of the central nervous system.. Mov Disord.

[18] Pizoli CE, Jinnah HA, Billingsley ML, Hess EJ (2002). Abnormal cerebellar signaling induces dystonia in mice.. J Neurosci.

[19] Eidelberg D, Moeller JR, Antonini A, Kazumata K, Nakamura T (1998). Functional brain networks in DYT1 dystonia.. Ann Neurol.

[20] Carbon M, Argyelan M, Eidelberg D (2010). Functional imaging in hereditary dystonia.. Eur J Neurol.

[21] Parent M, Levesque M, Parent A (2001). Two types of projection neurons in the internal pallidum of primates: single-axon tracing and three-dimensional reconstruction.. J Comp Neurol.

[22] Parent A, Sato F, Wu Y, Gauthier J, Levesque M (2000). Organization of the basal ganglia: the importance of axonal collateralization.. Trends Neurosci.

[23] Parent M, Parent A (2004). The pallidofugal motor fiber system in primates.. Parkinsonism Relat Disord.

[24] Blood AJ (2008). New hypotheses about postural control support the notion that all dystonias are manifestations of excessive brain postural function.. Biosci Hypotheses.

[25] Blood AJ, Tuch DS, Makris N, Makhlouf ML, Sudarsky LR (2006). White matter abnormalities in dystonia normalize after botulinum toxin treatment.. Neuroreport.

[26] Colosimo C, Pantano P, Calistri V, Totaro P, Fabbrini G (2005). Diffusion tensor imaging in primary cervical dystonia.. J Neurol Neurosurg Psychiatry.

[27] Fabbrini G, Pantano P, Totaro P, Calistri V, Colosimo C (2008). Diffusion tensor imaging in patients with primary cervical dystonia and in patients with blepharospasm.. Eur J Neurol.

[28] Oldfield RC (1971). The assessment and analysis of handedness: the Edinburgh inventory.. Neuropsychologia.

[29] van der Kouwe AJ, Benner T, Fischl B, Schmitt F, Salat DH (2005). On-line automatic slice positioning for brain MR imaging.. Neuroimage.

[30] Le Bihan D (2003). Looking into the functional architecture of the brain with diffusion MRI.. Nat Rev Neurosci.

[31] White T, Kendi AT, Lehericy S, Kendi M, Karatekin C (2007). Disruption of hippocampal connectivity in children and adolescents with schizophrenia–a voxel-based diffusion tensor imaging study.. Schizophr Res.

[32] Turkheimer FE, Smith CB, Schmidt K (2001). Estimation of the number of “true” null hypotheses in multivariate analysis of neuroimaging data.. Neuroimage.

[33] Blood AJ, Iosifescu DV, Makris N, Perlis RH, Kennedy DN (2010). Microstructural abnormalities in subcortical reward circuitry of subjects with major depressive disorder.. PLoS One.

[34] Breiter HC, Gollub RL, Weisskoff RM, Kennedy DN, Makris N (1997). Acute effects of cocaine on human brain activity and emotion.. Neuron.

[35] Aharon I, Etcoff N, Ariely D, Chabris CF, O'Connor E (2001). Beautiful faces have variable reward value: fMRI and behavioral evidence.. Neuron.

[36] Makris N, Gasic GP, Kennedy DN, Hodge SM, Kaiser JR (2008). Cortical thickness abnormalities in cocaine addiction–a reflection of both drug use and a pre-existing disposition to drug abuse?. Neuron.

[37] Bostan AC, Dum RP, Strick PL (2010). The basal ganglia communicate with the cerebellum.. Proc Natl Acad Sci U S A.

[38] Parent A (1996). Carpenter's Human Neuroanatomy; Rev. ed. of: Human neuroanatomy, Malcolm B. Carpenter, Jerome Sutin. 8th ed..

[39] Wenzel T, Schnider P, Wimmer A, Steinhoff N, Moraru E (1998). Psychiatric comorbidity in patients with spasmodic torticollis.. J Psychosom Res.

[40] Gundel H, Wolf A, Xidara V, Busch R, Ladwig KH (2003). High psychiatric comorbidity in spasmodic torticollis: a controlled study.. J Nerv Ment Dis.

[41] Heiman GA, Ottman R, Saunders-Pullman RJ, Ozelius LJ, Risch NJ (2004). Increased risk for recurrent major depression in DYT1 dystonia mutation carriers.. Neurology.

[42] Fabbrini G, Berardelli I, Moretti G, Pasquini M, Bloise M (2010). Psychiatric disorders in adult-onset focal dystonia: a case-control study.. Mov Disord.

[43] Stokely ME, Yorio T, King MA (2005). Endothelin-1 modulates anterograde fast axonal transport in the central nervous system.. J Neurosci Res.

[44] Ochs S, Pourmand R, Jersild RA, Friedman RN (1997). The origin and nature of beading: a reversible transformation of the shape of nerve fibers.. Prog Neurobiol.

[45] Haaland KY, Prestopnik JL, Knight RT, Lee RR (2004). Hemispheric asymmetries for kinematic and positional aspects of reaching.. Brain.

[46] Quartarone A, Pisani A (2011). Abnormal plasticity in dystonia: Disruption of synaptic homeostasis.. Neurobiol Dis.

[47] Gilio F, Curra A, Lorenzano C, Modugno N, Manfredi M (2000). Effects of botulinum toxin type A on intracortical inhibition in patients with dystonia.. Ann Neurol.

[48] Quartarone A, Morgante F, Sant'angelo A, Rizzo V, Bagnato S (2008). Abnormal plasticity of sensorimotor circuits extends beyond the affected body part in focal dystonia.. J Neurol Neurosurg Psychiatry.

[49] Tamura Y, Ueki Y, Lin P, Vorbach S, Mima T (2009). Disordered plasticity in the primary somatosensory cortex in focal hand dystonia.. Brain.

[50] Curra A, Trompetto C, Abbruzzese G, Berardelli A (2004). Central effects of botulinum toxin type A: evidence and supposition.. Mov Disord.

[51] Ruge D, Cif L, Limousin P, Gonzalez V, Vasques X (2011). Shaping reversibility? Long-term deep brain stimulation in dystonia: the relationship between effects on electrophysiology and clinical symptoms.. Brain.

[52] Ruge D, Tisch S, Hariz MI, Zrinzo L, Bhatia KP (2011). Deep brain stimulation effects in dystonia: Time course of electrophysiological changes in early treatment.. Mov Disord.

[53] Stavrinou LC, Boviatsis EJ, Stathis P, Leonardos A, Panourias IG (2011). Sustained Relief after Discontinuation of DBS for Dystonia: Implications for the Possible Role of Synaptic Plasticity and Cortical Reorganization.. Cen Eur Neurosurg.

[54] Finkelstein DI, Stanic D, Parish CL, Tomas D, Dickson K (2000). Axonal sprouting following lesions of the rat substantia nigra.. Neuroscience.

