# Supplementary material for: Evidence for Altered Basal Ganglia-Brainstem Connections in Cervical Dystonia
Source: PLoS One. 2012 Feb 22;7(2):e31654. doi: 10.1371/journal.pone.0031654 (PMC3285161; doi:10.1371/journal.pone.0031654)
Supplement: Methods S1 — This supporting information section describes the segmentation procedures used to define the search volumes for patient versus control contrast analyses. The first section describes the segmentation of a priori areas of evaluation (AOEs) for FA and MD contrasts, and the second section describes segmentation of the a priori search volume for the tractography contrast. (DOC) [file pone.0031654.s001.doc]

**Supporting Information Methods 1 (Methods S1). Description of segmentation procedures for contrast search volumes**

**Methods**

1. Segmentation of *a priori* areas of evaluation (AOEs) for FA and MD contrasts:

*A priori* AOEs were segmented by an anatomist (N.M.) with guidance from landmarks in neuroanatomic atlases [1,2,3] and localization of anatomical structures, which were visible on this average map. Segmentations were done directly on the average FA map for the healthy control group (registered to MNI standard space using the FSL FMRIB58_FA_1mm_brain template) (1) to ensure that segmentations were exactly registered with the maps being considered and (2) because the signal intensity contrast in the FA maps was better for delineating the subcortical anatomy than the T2 maps acquired as part of the DTI sequence. Examples of these segmentations are shown in Figure S1.

Segmentations were drawn with the intention of including all regions where a certain structure would be expected to fall, i.e. for use as a search volume. However, this did not preclude other tracts or structures from potentially falling within the region as well. Specifically, the individual anatomical structures, namely, the ansa lenticularis, substantia nigra, red nucleus, pedunculopontine nucleus, and the superior cerebellar peduncle were characterized as follows:

The ***ansa lenticularis*** (AL) is a set of fibers originating from the internal pallidal segment (GPi) (pp. 833-834 in [4]). These fibers rap around the posterior limb of the internal capsule as they course from the GPi towards their target structures. They first appear in the medial and lateral medullary lamina of the globus pallidus and then they bend under the ventral surface of the GPi maintaing a position between the GPi and the optic tract. Once it reaches the ventromedial tip of the globus pallidus (where it meets the lenticular fasciculus), the AL raps around the descending fibers of the corticospinal projection (at the posterior limb of the internal capsule) and penetrates the region under the zona incerta and gives some fibers to the subthalamic nucleus, red nucleus (rostral part), substantia nigra and the tegmental area. Progressing rostrally, it courses beneath the ventral anterior nucleus of the thalamus, to which it gives fibers. Finally, a small contingent of fibers passes between the mammilo-thalamic tract and the column of the fornix, which reaches the hypothalamus terminating in the posterior nucleus of the hypothalamus (pp. 35-37 in [5]). The AL shares a border ventrally with the optic tract with which gets into contact at the level of the internal capsule's genu. Dorsally the AL borders with the ventral surface of the GPi before it raps around the corticospinal tract. Laterally and caudally it borders with the corticospinal tract and medially with the hypothalamus (pp. 35-37 in [5]). Rostrally, the AL extends just behind the anterior commissure and the head of the caudate nucleus (pp. 422-423 in [6]). The AL first appears rostarlly in the coronal plane approximately 2mm behind the anterior commissure (AC) (coronal section 63 in (pp. 449-454 in [6]) occupying a territory within a triangular area defined by the ventromedial tip of GPi, the optic tract and the column of the fornix. Caudally, AL extends to the coronal plane of the mammilary body, which corresponds to 8 mm posterior to the AC or 5 mm posterior to the interventricular foramen of Monroe (pp. 140-152 in [7]). Thus it extends within a region of 7 mm within the rostrocaudal dimension. In the mediolateral dimension, AL appears in parasagittal planes starting at 7 mm (sagittal section 7 by (pp. 490-491 in [6]) or 8 mm (pp. 216-249 in [7]) from the mid-sagittal until 18mm (pp. 216-249 in [7]). Thus it is present within a tissue thickness of 11 mm. In the vertical dimension, AL appears first dorsally in an axial section 5mm above the AC (axial section 68, pp. 418-419 in [6]) and ventrally reaches a axial section 9 mm below the AC (axial section 73, pp. 422-423 in [6]). Thus it extends within a region of 5 mm within the vertical dimension. Guided by visual inspection and brain atlases as described above in detail, we segmented the AL in the MRI domain of the MNI 305 atlas, from coronal section 86 to (and including) coronal section 82 (Figure S1A).

The ***substantia nigra*** (SN) appears rostrally approximately 10-12 mm caudal to the AC and extends caudally at a location 3 mm caudal to the PC (fig. 59, p. 102 in [7]; [8,9]. Along its caudorostral course it is flanked laterally principally by the cerebral peduncle and medially by the red nucleus and the subthalamic nucleus. Medially the SN extends until 2 mm from the midline [8], laterally until 16 mm from the midline [8], dorsally it reaches 3 mm below the AC-PC line [8] and ventrally it reaches 13 mm below the AC-PC line [8]. Guided by visual inspection and brain atlases as described above in detail, we segmented the SN in the MRI domain of the MNI 305 atlas in 18 consecutive coronal sections to cover the entire SN in its anterior-posterior extent (Figure S1B). For the SN we also used the following three references: p. 4 and pp. 101-105 in [7], [9], and pp. 172-183 in [8].

The ***red nucleus*** (RN) is round-shaped and located medially to the SN, ventrally to the caudal part of the thalamus and laterally to the third ventricle. More rostrally, it borders with the caudal part of the subthalamic nucleus. In the rostrocaudal dimension it extends from the postrior commissure (PC) caudally to a coronal level at two thirds of the AC-PC distance proximal to the AC. Guided by visual inspection and brain atlases, we segmented the RN in the MRI domain of the MNI 305 atlas in 7 consecutive coronal sections (Figure S1C) [[6]; [9]; pp. 172-183 in [8]].

The ***pedunculopontine nucleus*** (PPN) is mostly located within the upper pons. The strongest landmarks for guidance are the middle cerebellar peduncle and the cerebellar decussation. The PPN appears to “hag” these fiber systems and they define its medial border [10]. The lateral border is half way between these fibers and the lateral exterior of the pons [10,11]. Guided by visual inspection and brain atlases [10,11], we segmented the PPN in the MRI domain of the MNI 305 atlas in 6 consecutive coronal sections (Figure S1D).

The ***superior cerebellar peduncle*** (SCP) delineation was done in the MRI domain of the MNI 305 atlas in 9 axial sections bilaterally. This was guided by the Angevine et al atlas (plate 406 L in [12]) and is shown in Figure S1E.

2. Segmentation of *a priori* search volume for tractography contrasts:

This segmentation included all regions that contain the pallidal output fibers of interest, i.e. the axon collaterals of the pallidothalamic fibers that project to the brainstem, as well as cerebellar white matter and deep nuclei (Figures S2-4). Because we did not want to limit our search volume to the hemisphere from which tractography was being computed, we used the entire bilateral segmentation as our search volume for both the left and the right pallidum tractography contrasts.

The dorsal boundary of the segmentation included all white matter several millimeters dorsal to the innermost point of the pallidum. The anterior boundary, superior to the brainstem, was the anterior-most portion of the putamen (this went anterior to the pallidum in order to be certain to include the region including the AL, which projects slightly anterior to the pallidum. The posterior boundary, superior to the brainstem, was the most posterior portion of the pallidum. Inferior to the pallidum, the segmentation covered the entire brainstem down through the pons. Within the cerebellum, all white matter and deep nuclei were included in the segmentation, but not cerebellar cortex.

**References**

1. Haines D (1991) Neuroanatomy: An atlas of structures, sections and systems.: Williams & Wilkins.

2. Nieuwenhuys R, Voogd J, van Huijzen C (1988) The Human Central Nervous System: A synopsis and atlas. Berlin: Springer-Verlag.

3. Mai JK, Assheuer J, Paxinos G (1997) Atlas of the Human Brain. New York: Academic Press.

4. Parent A (1996) Carpenter's Human Neuroanatomy; Rev. ed. of: Human neuroanatomy, Malcolm B. Carpenter, Jerome Sutin. 8th ed. C1983.: Lippincott, Williams, and Wilkins.

5. Delmas P (1959) Cranio-cerebral topometry in man. Springfield, Illinois: Charles C. Thomas.

6. Dejerine J (1895) Anatomie des Centres Nerveux. Paris: Rueff et Cie.

7. Andrew J, Watkins E (1969) A stereotaxic atlas of the human thalamus and adjacent structures, pp. 140-152. Baltimore: Williams and Wilkins.

8. Talairach J, David M, Tournoux P, Corredor H, Kvasina T (1957) Atlas d'Anatomie Stereotaxique des Noyaux Gris Centraux. Paris: Masson et Cie.

9. Talairach J, Tournoux P (1988) Co-planar stereotaxic atlas of the human brain. New York, NY: Thieme Medical publishers.

10. Olszewski J, Baxter D (1954) Cytoarchitecture of the Human Brain Stem. Basel and New York: S. Karger.

11. Paxinos G, Huang X (1995) Atlas of the Human Brainstem. San Diego, CA: Academic Press.

12. Angevine J, Mancall E, Yakovlev P (1961) The Human Cerebellum: An Atlas of Gross Topography in Serial Sections. Great Britain: J&A Churchill Ltd.
