# Supplementary figures and images for: Evidence for Altered Basal Ganglia-Brainstem Connections in Cervical Dystonia
Source: PLoS One. 2012 Feb 22;7(2):e31654. doi: 10.1371/journal.pone.0031654 (PMC3285161; doi:10.1371/journal.pone.0031654)

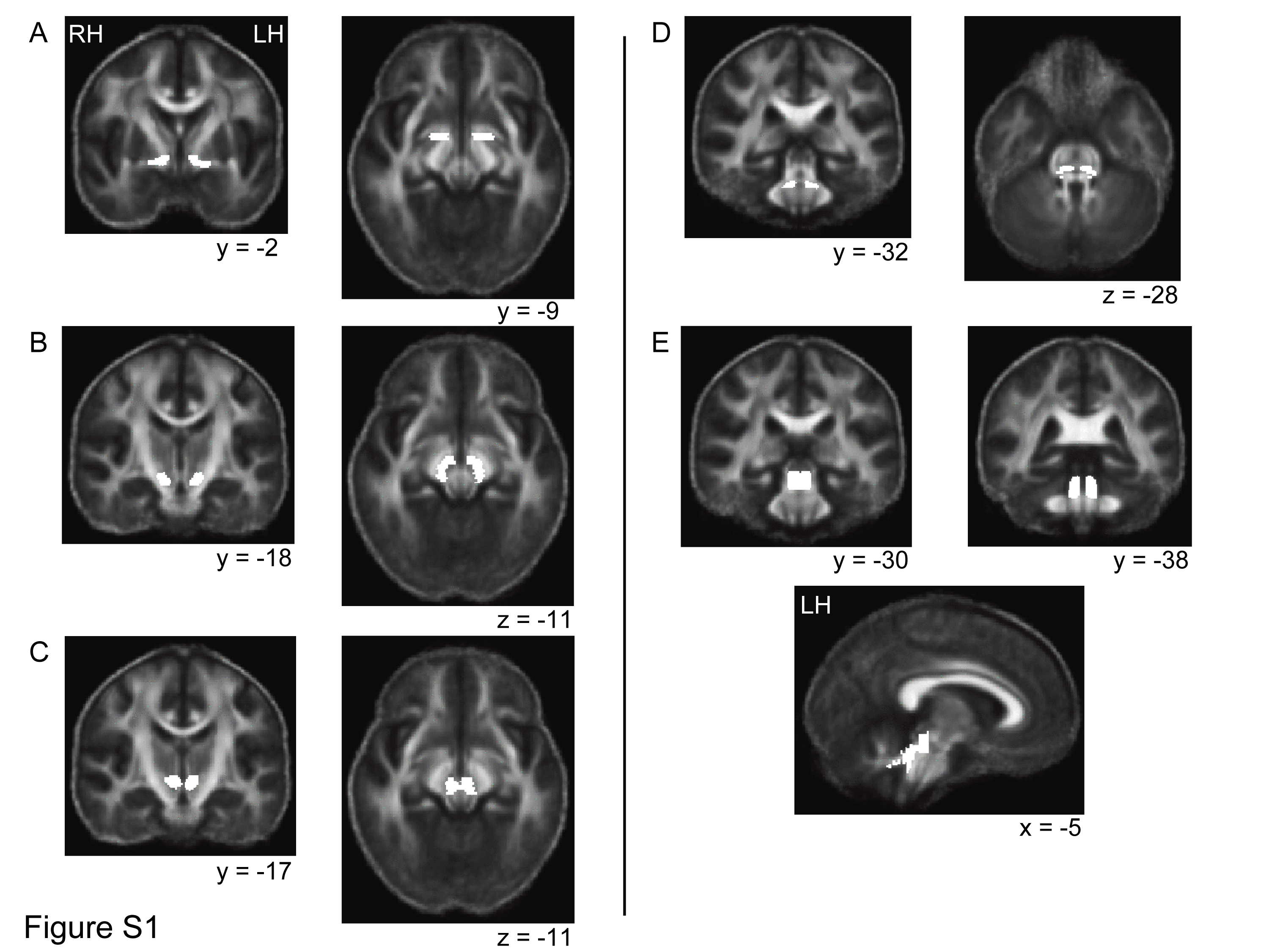

Supplement: Figure S1 — Examples of segmentations for each a priori area of evaluation (AOE) used, collectively, as the search volume in the FA and MD contrasts. Segmentations are shown in white, superimposed on the average FA brain for all 24 subjects in the study. MNI coordinates are indicated for each image. (A) Segmentation of the ansa lenticularis (AL) (B) Segmentation of the substantia nigra (SN) (C) Segmentation of the red nucleus (D) Segmentation of the pedunculopontine nucleus (E) Segmentation of the superior cerebellar peduncle. (TIF) [file pone.0031654.s002.tif]

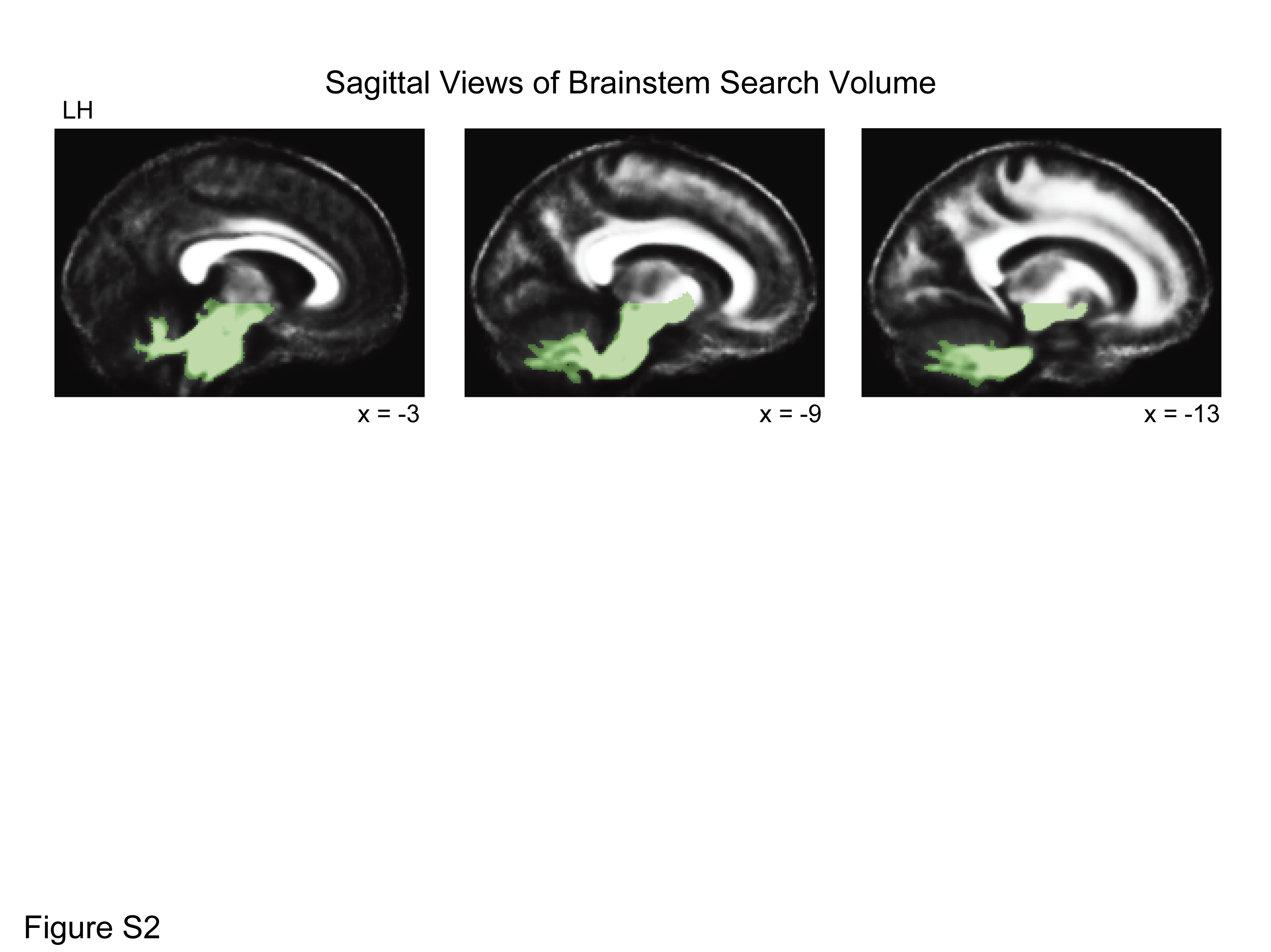

Supplement: Figure S2 — Examples of the segmentation used as the a priori search volume in the tractography contrasts, shown from a sagittal view. MNI talairach coordinates are indicated for each image. LH: left hemisphere. (TIF) [file pone.0031654.s003.tif]

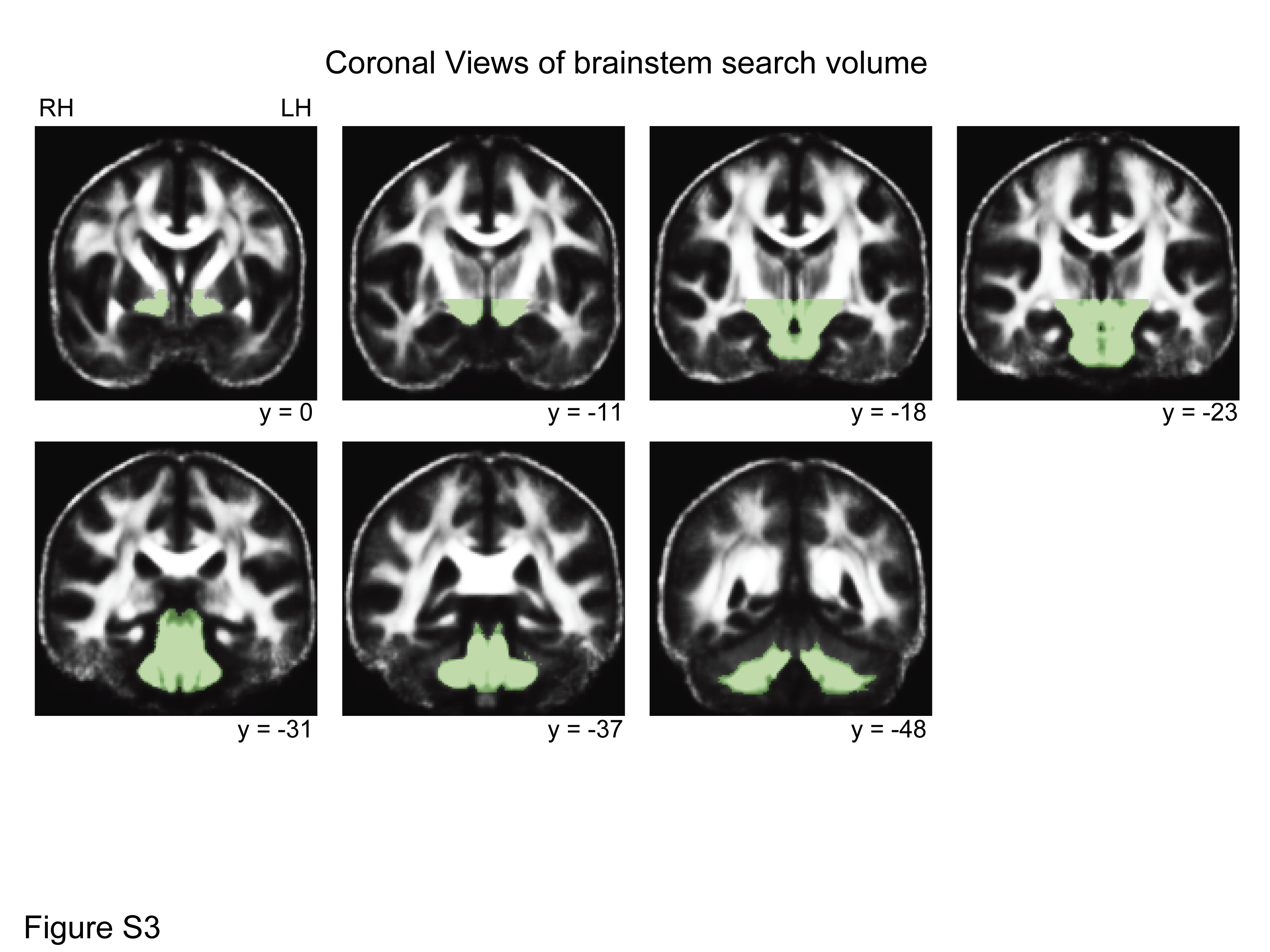

Supplement: Figure S3 — Examples of the segmentation used as the a priori search volume in the tractography contrasts, shown from a coronal view. MNI talairach coordinates are indicated for each image. LH: left hemisphere; RH: right hemisphere. (TIF) [file pone.0031654.s004.tif]

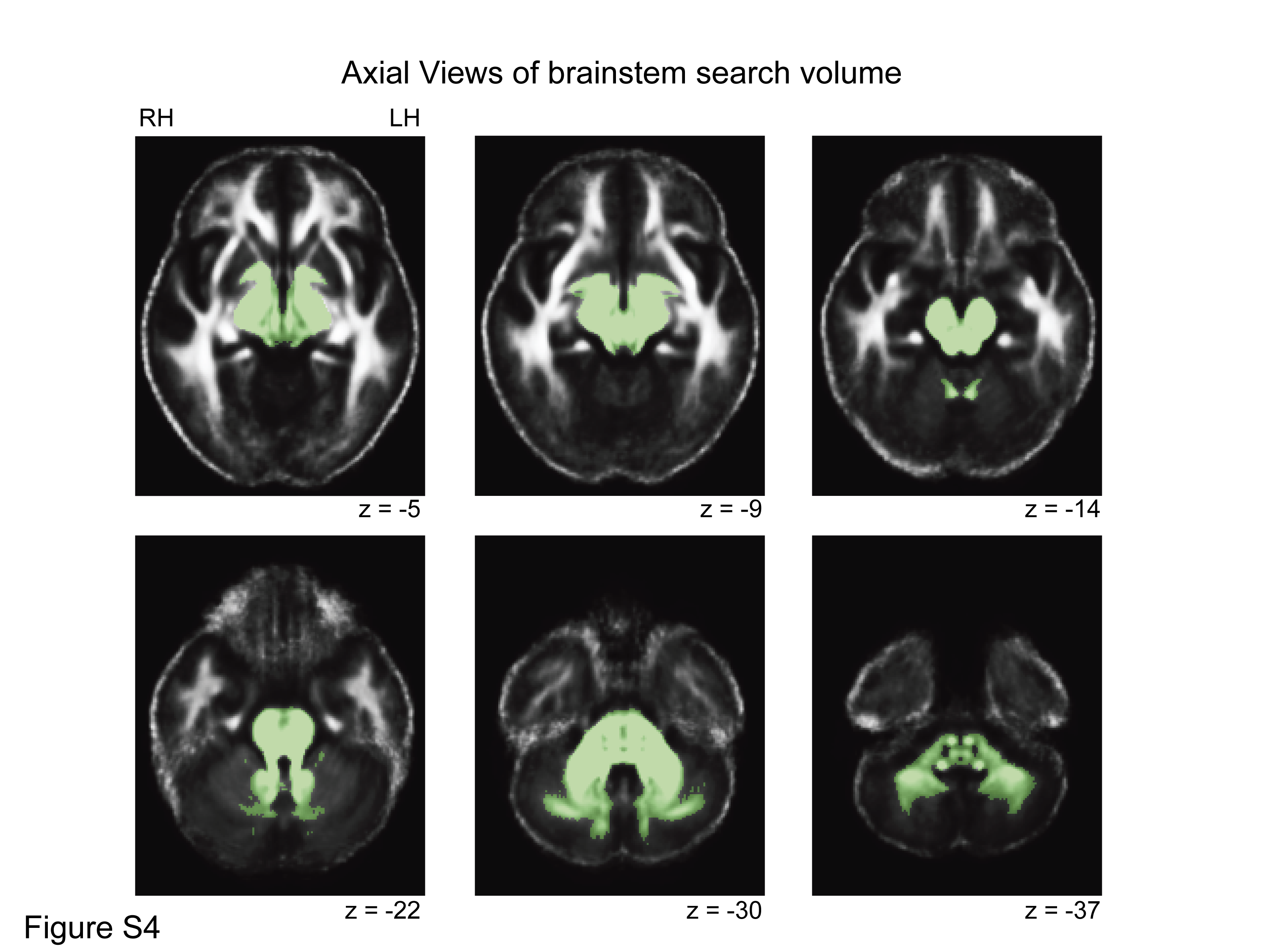

Supplement: Figure S4 — Examples of the segmentation used as the a priori search volume in the tractography contrasts, shown from an axial view. MNI talairach coordinates are indicated for each image. LH: left hemisphere; RH: right hemisphere. (TIF) [file pone.0031654.s005.tif]

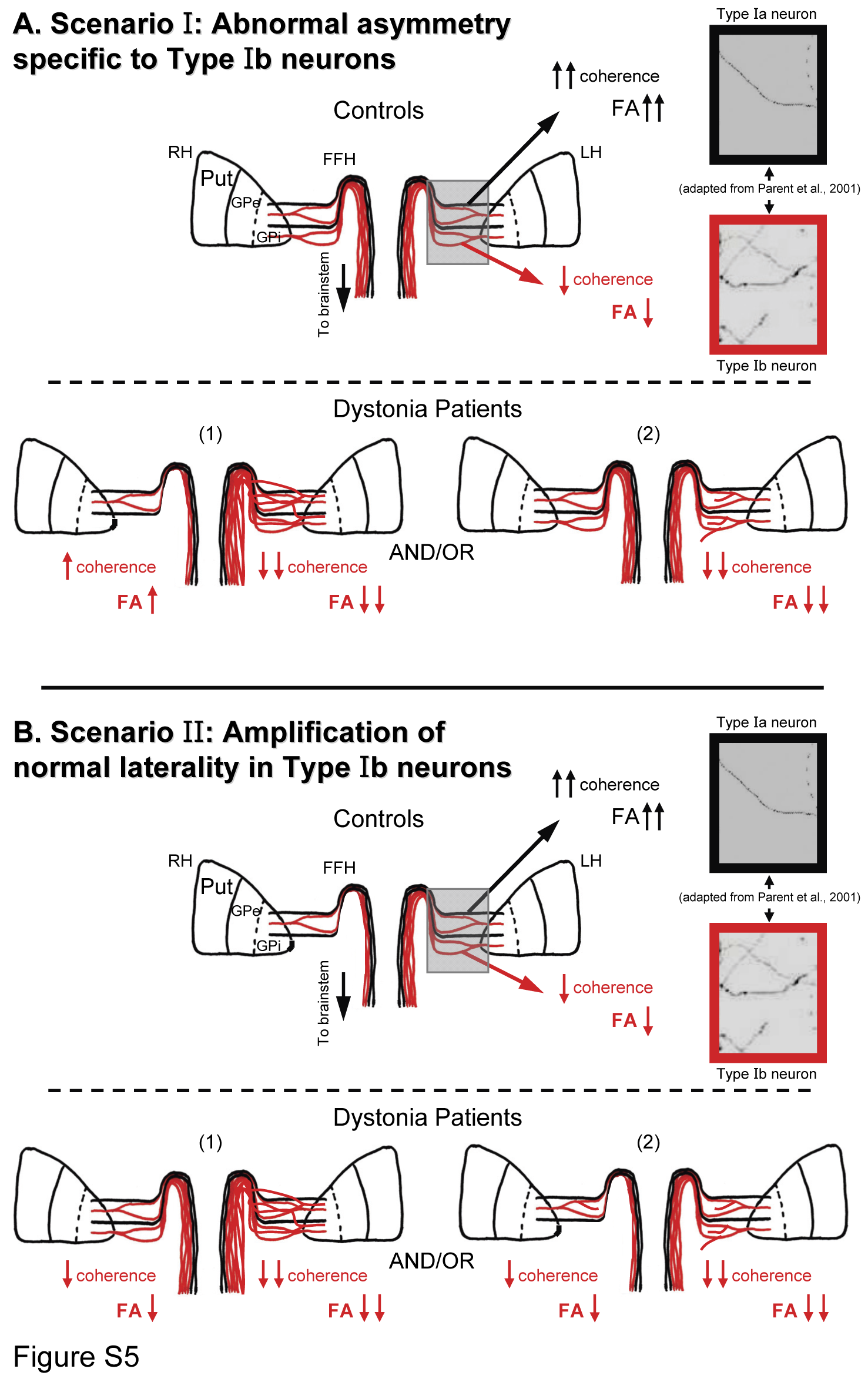

Supplement: Figure S5 — This figure suggests some theoretical ways in which two primary types of motor neurons projecting from the pallidum [21] might influence diffusion tensor imaging (DTI) measures. The primary issue at hand is that one fiber type, Type Ib, exhibits much more extensive collateralization than the other (Type Ia), even within the region immediately medial to the pallidum, and that collateralization reduces axon coherence. Given that axon coherence is a major component influencing fractional anisotropy (FA), if the relative proportion of these fibers is increased, FA will decrease. Because anisotropy influences probabilistic tractography, tractography will also be reduced if the relative proportion of these fibers is increased, perhaps even if there are more fibers projecting through the region. (A) Shows the effects that collateralization will have on FA, with examples of how these effects might be altered if the relative numbers of fibers in the left versus right hemispheres (1) or the amount of collateralization from existing fibers (2) increases or decreases in dystonia. While (1) is more likely to develop from asymmetric loss of neurons (less likely in primary dystonia), it is not out of the question that (2) could take place as a microstructural response to changes in function, given that terminal arborization has been shown to take place over weeks [54]. (B) Suggests the possibility that healthy individuals exhibit asymmetries in the left/right proportions of the Type Ib fibers relating to normal motor laterality, which only become detectable with DTI when there is a relative increase in the number of these fibers in proportion to other fibers in this region that collateralize less (e.g. Type Ia fibers), or not at all (descending internal capsule fibers). (TIF) [file pone.0031654.s006.tif]
